# AGE AND ADVANCE CARE PLANNING PREDICTS SELF-PERCEIVED RISK FOR DEMENTIA

**DOI:** 10.1093/geroni/igz038.1735

**Published:** 2019-11-08

**Authors:** Stacy W Yun, Molly Maxfield

**Affiliations:** University of Colorado Colorado Springs, Colorado Springs, Colorado, United States

## Abstract

Self-perceived Alzheimer’s disease and related disorders (ADRD) risk may be highly influenced by the sense of control which one has over the prevention and negative impact of such diagnoses (Kessler et al., 2012). This study examined whether age, advance care planning (ACP), and experimentally manipulated dementia salience (DS) are related to self-perceived risk of dementia. Participants (N = 122, 40 to 88 years old, M = 65.66, SD = 9.71) completed the computerized study using Qualtrics software. Participants completed questionnaires assessing individual differences, self-perceived risk, and demographics. Multiple regression was calculated to predict self-perceived ADRD risk based on age, ACP, and DS induction. The set of predictors explained 15.3% of variance in participants’ self-perceived risk of dementia, F(3, 118) = 7.08, p < 0.001. Specifically, being older (β = -0.40, p < 0.001) and having less ACP items completed (β = 0.26, p < 0.01) uniquely predicted lower perceived risk. DS condition (β = -0.01) did not attain significance in the model. Younger age might be associated with less ADRD exposure and understanding, which may influence perceived risk. It is possible that older adults are less worried about developing dementia as they may (erroneously) believe they have “passed” the time to develop ADRD. Additionally, high levels of self-perceived ADRD risk may have resulted in motivation for greater levels of planning for the future among those with greater ACP. Future studies will investigate the level or point in which perceived risk changes from being beneficial and health-promoting to problematic and anxiety-provoking.

